# Study on the correlation between basketball players’ multiple-object tracking ability and sports decision-making

**DOI:** 10.1371/journal.pone.0283965

**Published:** 2023-04-05

**Authors:** Qifeng Gou, Sunnan Li

**Affiliations:** 1 College of Physical Education, Northwest Normal University, Lanzhou, Gan Su, China; 2 College of Physical Education and Sports, Beijing Normal University, Beijing, China; University of Split, CROATIA

## Abstract

**Background:**

Players’ multiple-object tracking (MOT) ability is very important in basketball because it may affect players’ sports decision-making (SDM), thus affecting the results of the game. The purpose of this study was to investigate the differences between expert and novice basketball players in MOT ability and SDM and to explore the correlation between basketball players’ visual attention and SDM.

**Methods:**

A total of 48 female basketball players (24 categorized in the expert group and 24 in the novice group) participated in the MOT task in Experiment 1 and the basketball 3 vs. 3 games in Experiment 2. Experiment 1 examined the difference in dynamic visual attention characteristics between expert players and novice players by changing the tracking number. Experiment 2 examined the differences between expert players and novice players through the SDM of basketball 3 vs. 3 games. Sports decisions were evaluated by basketball experts. MOT ability and SDM ability were analyzed through Pearson correlation.

**Results:**

The overall MOT accuracy of expert players (64.6%) and novice players (55.7%) was significantly different (*χ*^*2*^ = 59.693, *P* = 0.000). There was no significant difference in accuracy when tracking 2–3 targets (*P* > 0.05), but there was a significant difference in accuracy when tracking 4–6 targets (*P* < 0.05). The overall SDM accuracy of expert players (91.6%) and novice players (84.5%) was significantly different (*χ*^*2*^ = 31.975, *P* = 0.000). There was no significant difference between expert players and novice players in the accuracy of dribbling decision-making (*P* > 0.05), but there was a significant difference in the accuracy of passing decision-making and shooting decision-making (*P* < 0.01). When tracking 4–5 targets, the tracking score was positively correlated with the passing decision score and dribbling decision score of expert players, and the tracking score of novice players was positively correlated with the passing decision score (*r* > 0.6, *P* < 0.01).

**Conclusions:**

First, the tracking accuracy of expert players was significantly higher than that of novice players, especially when tracking 4–6 targets. As the number of targets increased, accuracy decreased. Second, the accuracy of expert players’ SDM was significantly higher than that of novice players, especially in passing decision-making and shooting decision-making. Expert players exhibited fast and accurate SDM. Third, there was a correlation between MOT ability and SDM performance. The MOT ability of 4–5 targets was positively correlated with passing decision-making, which was statistically significant. The correlation between the MOT ability and SDM performance of expert players was greater and more significant. Having too many targets to track (more than 6) interfered with players’ decisions.

## Introduction

"Decision" means consciously choosing from different possible actions [1]. Players’ professional sports skills and speed are closely related to high-level performance in visual attention tasks [2]. In particular, the ability to make decisions under time pressure is crucial to the results of a match [3]. Efficient visual attention and rapid working memory processing at key nodes of the competition are the prerequisites for making accurate decisions during a match [4]. The first step of sports decision-making (SDM) is perception; neurons transmit information to the central nervous system that enable a person to make decisions [5]. From the perspective of operation, the dynamics of perception, action and cognition can be described at two levels of analysis [6]. The first-level analysis determines information variables that guide behavior [7]. The second-level analysis characterizes the time evolution of this behavior [8]. The effectiveness of SDM refers to whether the decision-making performance conforms to the current situation and solves problems [5]. Sanchez et al. used questionnaires and interviews to study how high-level female basketball players make decisions in competitions, and the results showed that players are experts in making decisions during competitions [1]. More research required subjects to watch images or videos about which they would have to make decisions to reflect the accuracy of SDM, and the results showed that players made faster and more accurate decisions after viewing them [9], but there are few studies on on-site sports decisions. An eye tracker was used to show which areas players prefer to focus on when making sports decisions (e.g., an open area without offensive and defensive players [10]). Some studies have also used electroencephalogram (EEG), functional near-infrared spectroscopy (fNIRS) and functional magnetic resonance imaging (fMRI) techniques, and the prefrontal cortex plays governs motor decision-making [11]. An increasing number of studies have taken attention control as a driving factor for the limitation of visual short-term and working memory ability [12]. The ability to remember previously seen information in a short time is critical to many cognitive and perceptual processes and is called visual working memory (VWM) [12]. More than 80% of brain information comes from visual attention [13]. Good attention quality is key for players to effectively implement sports skills; it helps players to more effectively and selectively pay attention to, recognize and interpret visual information and provides sufficient information for further decision-making and responses [14]. In ball games, when and how long do players turn to other more important clues? The ability to predict the direction and speed of the ball according to clues provided by the opponent’s action is often the key to victory [5]. Team ball games require players to be able to pay attention to the changes in multiple teammates, opponents, and their positions at the same time; therefore, multiple-object tracking (MOT) ability is very important for playing team ball games [15]. Improving the attention ability of basketball players to promote the accuracy of players’ decision-making has become the focus of coaches and sports research experts in various countries [10].

We need to pay attention to and track the motion of multiple objects at the same time, a process referred to as MOT [16]. MOT combines the selectivity, limited capacity and subjective effort of attention and can reflect multiple aspects of attention, such as selective attention, distributive attention, and continuous attention [17]. MOT performance is affected by many factors, such as the number of targets and nontargets [18] and the speed of object movement [19]. The research method used most is to divide the subjects into expert and novice groups and guide their training to identify the gap between the expert and novice groups [20]. Mangine et al. conducted research on 12 National Basketball Association (NBA) players using 3D MOT tasks and found that players with good visual tracking speed performed better in games [21], and football players’ visual intervention improved SDM [22]. Rugby defenders performed better than offensive players and college students in MOT tasks [23]. With an increasing number of targets, the tracking performance of the basketball players in the expert group was better than that in the novice group, the tracking performance of the guard was the best, and those playing the center position performed slightly better than those in the forward position [24]. Qiu et al. found that compared with intermediate or nonplayers, excellent basketball players showed better tracking performance when tracking 3 or 4 targets. However, no significant differences were found between intermediate players and nonplayers. In addition, no difference was observed among the three groups when tracking 2 targets [25]. When the number of targets increased to 6 targets, basketball players had better tracking performance than college students who did not play sports [2]. Maarseveen et al. studied the sports decisions of high-level basketball players in situ of specific 3 vs. 3 basketball matches and found that players began to check possible choices earlier when deciding to shoot or pass the ball than when making breakthroughs [26]. Skilled players aimed at the ball holder when defending and used peripheral vision to observe the movement of other players [27].

However, there are few studies on female subjects [28], yet some scholars found that expert players had no obvious advantage in MOT ability [20], team players in specific positions had no advantage in MOT tasks [29], and 3D MOT training did not significantly improve SDM [30,31]. More research supports that professional players have better MOT ability [17,32]. The ability of players to track multiple targets may be related to long-term skill training [10,33]. 3D MOT and decision-making task performance are related [34]. 3D MOT task training improves the decision-making accuracy of football players [33]. However, few people have studied the relationship between the number of items tracked during MOT tasks and SDM [10]. Therefore, this study used the expert-novice paradigm to verify the relationship between the number of MOT tasks tracked and SDM.

This study assessed the relationship between MOT ability and SDM. Experiment 1 used MOT tasks to compare the dynamic visual attention characteristics of female expert and novice basketball players. Experiment 2 compared the SDM of female expert and novice basketball players. Then, the relationship between the dynamic visual attention characteristics of basketball players and SDM was evaluated. We hypothesized that female expert basketball players’ MOT ability and SDM would be better than those of novice players and that there is a correlation between MOT ability and SDM ability. The correlation between the accurate score of MOT and the accurate score of SDM of expert players and novice players is different; therefore, it may be possible that the correlation of expert players is greater.

## Materials & methods

### Participants

This research used G*Power 3.1.9.7 (Germany) software to estimate the sample size and set the effect size as ηp2 = 0.03 and two-tailed α = 0.05 [35], and a statistical power of 0.80 was reached for 40 subjects [36]. Controlling for attrition, we selected 48 subjects (24 in each group) [2]. Forty-eight subjects were divided into either an expert group or a novice group according to their basketball experience level. The expert players were selected from the Northwest Division of the China University Basketball League Division One [10]. The age range of expert players was 18–22 years (mean: 20.31; SD: 2.25 years), with more than 9 years of experience per player (mean: 9.78; SD: 2.43 years) and over 15 hours (mean: 15.02; SD: 2.86 hours) of training per week in the past year. The novice group consisted of 24 students from the sports basketball elective course of Northwest Normal University, aged 18–21 years (mean: 19.35; SD: 1.62 years), who had not participated in basketball training. All participants were female, right-handed, with normal or corrected vision, and the average game video time per week did not exceed 4 hours [37]. All subjects participated in the experiment and received compensation for their time. The study was approved by the Ethics Committee of Northwest Normal University (No. 20210812). All of the participants provided written informed consent prior to the start of the experiment.

### Apparatus

In Experiment 1, a Thinkpad E15 laptop was used. The program environment was Windows 10, the display screen was 15.6 inches (34.4 cm × 19.4 cm), the screen resolution was 1920 × 1080 pixels, and the refresh rate was 60 Hz. MATLAB R2020b (The MathWorks Inc., Natick, MA, USA) software and Psychtoolbox 3 (version: 3.0.17) were used to program the MOT experiment. At the beginning of the experiment, a "+" symbol appeared in the middle of a gray background (38.20°× 21.3°) for 1000 ms. Then, the screen displayed 12 white balls (0.66° in diameter), of which 2–6 balls changed from white to blue, flashed 3 times (2 s) and were marked as targets. The balls that did not flash were considered as nontargets. After that, all the balls returned to their original white color. Then, the balls started to move independently at a speed of 5°/s. During this process, there may have been occlusion between the balls. After 10 s, the balls stopped moving, and the same number of balls as the target were randomly selected and marked in red. The subject needed to judge how many targets were marked red, press the corresponding number key, and then begin the next test (e.g., in a 4-target condition, there were 4 randomly selected items in red at the end of tracking time, and the subject had to determine if any of them were actual targets; then, participants had to press keys corresponding to 0–4).

In Experiment 2, two cameras (Canon, HFG50) were used to record the basketball 3 vs. 3 match. The camera was placed on the auditorium of the stadium, approximately 10 meters away from the competition site, such that it could capture the entire competition area. Players were identified by their jerseys and numbers, and the video was analyzed by Storm Video software (version: 5.81.0202.1111).

### Design

In Experiment 1, the MOT task was used to examine the tracking performance under different target numbers. A mixed experimental design of 2 (groups) × 5 (number of targets) was used. The groups (experts and novices) were the variables between the subjects. The number of targets (2–6) was an intrasubject variable, and the dependent variable was the tracking accuracy. The accuracy algorithm was defined as the percentage that each subject correctly selected the target in all experimental trials. In Experiment 2, the basketball 3 vs. 3 match was judged by three fixed basketball referees. SDM was evaluated by two basketball experts according to the game video and coding standards [33]. For the evaluation results with objections, a third basketball expert was invited to discuss and assess. Before the experiment, 5 other experts unanimously agreed that the coding standard was valid. One point represented an accurate decision, 0 points represented an inaccurate decision, and decisions that were both accurate and inaccurate were not coded. To avoid the impact of rapid responses on accuracy, we did not require subjects to respond quickly to ensure accuracy [25]. The experiment was accompanied by 1 tester to ensure completion.

### Procedure

**Fig 1:**
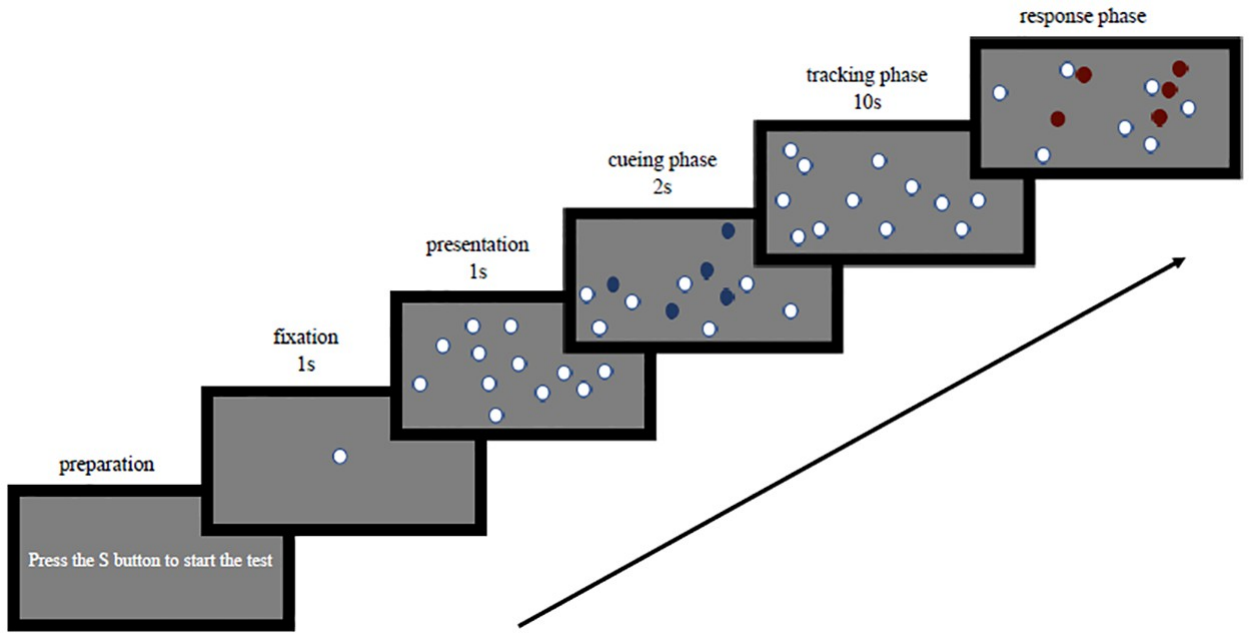
MOT task process.

Experiment 1 was conducted in the laboratory of Northwest Normal University from August 25, 2021, to August 28, 2021. To familiarize the subjects with the experimental process, there were 8 preexperiments before the formal experiment. The distance between the subjects and the screen was approximately 60 cm. The experiment was composed of 5 blocks, each of which consisted of 2–6 targets. Each block had 30 trials, for a total of 150 trials. In each block, a white screen was displayed for 1 minute in the middle to alleviate participant eye fatigue. The experimental sequence was balanced within the subjects, and the whole experiment lasted approximately 27 minutes. The experimental process is shown in Fig 1.

The basketball 3 vs. 3 match in Experiment 2 was held in the Basketball Hall of Northwest Normal University from August 29, 2021, to September 1, 2021. Before the experiment, all subjects were asked to know the rules of 3 vs. 3 matches very well. Participants were randomly assigned to 8 teams in the same group, and each team was composed of 3 participants. Eight teams met randomly in 28 matches (no two teams met twice), and each match was 5 minutes. During the entire test, each person participated in 7 games, totaling 35 minutes.

### Statistical analysis

The data were recorded in and collected by MATLAB R2020b software. SPSS 26.0 (SPSS, Inc., Somers, New York, USA) software was used to compare the differences in MOT ability and SDM performance and evaluate the correlation between MOT ability and SDM across the two groups. Pearson’s χ-squared test was used to compare the accuracy of the MOT and SDM performance, and Pearson’s correlation coefficient was used to compare the correlation between the MOT and SDM performance. An ɑ level of 0.05 was considered statistically significant for all of the statistical comparisons. SDM ability was coded using standardized tools [33,38]. Then, the scores of each player in each game were added to calculate the total score. The correlation was calculated by the SDM accuracy and the accuracy for the MOT task. The scoring criteria were as follows: 1 point for each accurate result and 0 points for each inaccurate result [33].

## Results

### Accuracy of visual tracking number trials (AVTNs)

Pearson’s χ-squared test results of Experiment 1 showed that there was a significant difference in the total MOT accuracy between expert players (64.6%) and novice players (55.7%) (*χ*^*2*^ = 59.693, *P* = 0.000). When tracking 2–6 targets, the accuracy of expert players was higher than that of novice players, and the accuracy decreased with increasing tracking load. There was no significant difference in accuracy when tracking 2–3 targets (*P* > 0.05), but there was a significant difference in accuracy when tracking 4–6 targets (*P* < 0.05), as shown in Table 1.

**Table 1:** Comparison of AVTNs between the expert group and novice group.

Tracking load	Accurate (%)		*χ* ^*2*^	*P* value
	Expert group	Novice group		
2 targets	672 (93.3)	654 (90.8)	3.086	0.079
3 targets	576 (80.0)	546 (75.8)	3.632	0.057
4 targets	432 (60.0)	384 (53.3)	6.516	0.011*
5 targets	405 (56.3)	324 (45.0)	18.228	0.000**
6 targets	240 (33.3)	96 (13.3)	80.497	0.000**
Total	2325 (64.6)	2004 (55.7)	59.693	0.000**

Notes.

* *P* < 0.05.

** *P* < 0.01.

### Accuracy of SDM trials (ASDMs)

In Experiment 2, the Pearson χ-squared test results showed that the overall SDM accuracy of expert players (91.6%) and novice players (84.5%) was significantly different (*χ*^*2*^ = 31.975, *P* = 0.000). The overall decision-making speed of the expert players was faster than that of the novice players (0.74 times per second vs. 0.52 times per second, respectively), and the accuracy of the expert players in the three techniques of passing, dribbling and shooting was higher than that of the novice players. There was no significant difference in the accuracy of dribble decision-making between expert players and novice players (*P* > 0.05). The study showed that the number of times dribble decision-making occurred was greatly different across the three techniques, and the expert players had more than twice as many decisions resulting in dribbling as the novice group (658 times vs. 278 times, respectively). There were also significant differences between passing decision-making and shooting decision-making accuracy (*P* < 0.01), as shown in Table 2.

**Table 2:** Comparison of ASDMs between the expert group and novice group.

Decision criteria	Accurate (%)		*χ* ^*2*^	*P* value
	Expert group	Novice group		
Passing	457 (96.4)	399 (89.3)	17.938	0.000**
Dribbling	624 (94.8)	263 (94.6)	0.021	0.886
Shooting	339 (80.9)	256 (70.7)	11.112	0.001**
Total	1420 (91.6)	918 (84.5)	31.975	0.000**

Notes.

** *P* < 0.01.

### Pearson correlation between MOT ability and SDM performance

Pearson’s correlation coefficient between the accuracy score of visual tracking number trials (SVTNs) and the accuracy score of SDM (SSDMs) was calculated [2]. The size of each correlation coefficient used the following evaluation criteria: < 0.1 = trivial; 0.1–0.3 = small; 0.3–0.5 = moderate; and 0.5–0.7 = large [39]. The analysis showed that when tracking 2 targets, there was a medium positive correlation between the SVTN and the shooting SSDM of expert players (r = 0.403, P = 0.051), and there was no correlation between the SVTN and the shooting SSDM of novice players (*r* = -0.018, *P* = 0.935). When tracking 3 targets, there was a medium negative correlation between the SVTN and expert players’ dribbling SSDM (*r* = -0.307, *P* = 0.144), and there was no correlation between the SVTN and novice players’ dribbling SSDM (*r* = 0.043, *P* = 0.843). When tracking 4–5 targets, the SVTN was positively correlated with the passing SSDM and dribbling SSDM of expert players, and the SVTN was positively correlated with the passing SSDM of novice players, which was statistically significant (*r* > 0.6, *P* < 0.01), indicating that the tracking ability of a large number of targets is significantly correlated with accurate passing decisions. When tracking 6 targets, for expert players, there was a small negative correlation between the SVTN and dribbling SSDM and between the SVTN and shooting SSDM (0.1 < *r* <0.3, *P* > 0.05), and for novice players, there was a small negative correlation between the SVTN and passing SSDM, between the SVTN and dribbling SSDM and between the SVTN and shooting SSDM (0.1 < *r* < 0.3, *P* > 0.05). This shows that tracking with more than 6 targets has an interference effect on SDM, as shown in Table 3.

**Table 3:** Correlation between MOT ability and SDM performance among basketball players.

Target Number	Pearson correlation	Expert group			Novice group		
		Passing	Dribbling	Shooting	Passing	Dribbling	Shooting
2 targets	*r* value	-0.235	-0.197	0.403	0.145	0.131	-0.018
	*P* value	0.270	0.357	0.051	0.499	0.541	0.935
3 targets	*r* value	-0.140	-0.307	-0.206	0.100	0.043	0.088
	*P* value	0.513	0.144	0.334	0.642	0.843	0.681
4 targets	*r* value	0.711**	0.704**	0.089	0.785**	0.215	-0.008
	*P* value	0.000	0.000	0.679	0.000	0.313	0.970
5 targets	*r* value	0.667**	0.666**	-0.056	0.724**	0.104	-0.016
	*P* value	0.000	0.000	0.794	0.000	0.628	0.941
6 targets	*r* value	0.005	-0.191	-0.231	-0.199	-0.203	-0.193
	*P* value	0.982	0.370	0.277	0.350	0.341	0.367

Notes.

** *P* < 0.01.

## Discussion

The purpose of this study was to investigate the differences between visual attention and SDM among female expert and novice basketball players and determine the correlation between visual attention and SDM. The results verified our hypothesis. Compared with novice players, expert players had advantages in visual attention and SDM, and visual attention and SDM were related. In the dynamic movement of team ball games, the ability to "read the game" distinguishes skilled players from unskilled players [40]. Expectations and decisions represent the ability of the human brain to extract meaningful contextual information from visual scenes and are crucial for high-level sports performance [33]. Eye movement control depends on two interactive yet distinct processes: attentional decision-making to allocate value to information sources and SDM to flexibly link selected information with actions [41]. Physical activity is generally believed to enhance brain plasticity and improve cognitive and executive functions. Experts have been proven to be superior to subelite and novice players in sports-specific tasks, including the utilization of advanced visual cues [42], visual search strategies [10], anticipation and decision-making skills [5]. Voss and his colleagues showed that professional knowledge of sports is related to high-level performance in processing speed and visual attention [28]. The superior perception of more highly skilled players may be attributed to their skills or abilities, rather than their greater experience or exposure to tasks [40]. Our research results showed that when tracking 2–6 targets, the tracking accuracy of expert players was significantly higher than that of novice players (64.6% vs. 55.7%, respectively). With an increasing number of visual tracking objects, the accuracy of tracking objects decreased among all participants. This highlights that the capacity of attention resources is limited; as the number of targets increases, the resources allocated to each target decrease, which leads to a decline in tracking accuracy [18]. When tracking 4–6 targets, the accuracy of the expert players was significantly higher than that of the novice players, and the expert players had an MOT advantage. This finding supported the research results of Qiu and Li [11,24,25] but is different from the research results of Jin and Ji [2,20]. The visual search strategy involved in the MOT process may be closely related to the strategy of motion experts in extracting visual information from actions [33]. In basketball, players monitor the movements and positions of other players (teammates and opponents) to quickly make good passes, which is similar to tracking multiple objects in MOT [2]. When players have to navigate 1 vs. 1 and 2 vs. 2 situations, they feel confident and need to "read" the defense before deciding what to do [1]. The expert players exhibited faster and higher accuracy in SDM when compared to novice players (0.74 times per second vs. 0.52 times per second, 91.6% vs. 84.5%, respectively). The study showed that the number of times dribble decision-making occurred had the greatest difference across the three techniques, and the expert players had more than twice as many as the novice players (658 times vs. 278 times, respectively). This outcome may be due to the low dribbling skills of novice players. On-court performance is the result of the combination of SDM and technical skill level. SDM is the premise, technical level is the basis, and on-court performance is the result [5].

Our study found that when tracking 4–5 targets, the SVTN was positively correlated with passing SSDM, and the correlation was statistically significant. When tracking 4–5 targets, the SVTN of the expert players and novice players was positively correlated with the passing SSDM, and the SVTN of the expert players was larger and more significant. These findings reflect the visual attention advantage of expert players. When tracking 6 targets, the SVTN was negatively correlated with SSDM, which indicated that having to attend to too many tracking targets (more than 6) interfered with players’ decisions. This finding is different from some research results [29,30]. Good visual attention is a key component of players’ accurate SDM, and it is more obvious in high-level competitions. In the 3 vs. 3 competition, the number of perceptual information sources is not so high, and the role of peripheral vision is more emphasized to distribute attention more evenly according to specific situations [40]. This tells us that attention training should be applied to physical education teaching and training. Compared with dribbling and shooting, 3D MOT training can significantly improve players’ passing decisions [33]. Ten sessions of 3D MOT training improved attention, visual information processing speed and working memory. In addition, 3D MOT training can be transferred to social-related tasks [43]. Neurological studies have demonstrated the role of 3D MOT training in enhancing the cognitive function of healthy young people [44]. MOT technology reported the activation of brain regions involved in the attention process [45]. These regions include the parietal and frontal lobe regions of the cortex, which are considered to be the regions responsible for attention shifts and eye movement [46]. When reading a situation during a competition, players activate the movement perception process in the dorsal and ventral areas [47]. Attention tracking of multiple elements during 3D MOT training may overlap with the brain network required in the decision-making process. Future imaging or EEG research will help us explain this neural process [33].

This study provided an effective reference for the transfer of SDM from the laboratory to the live situation and solves the correlation between visual attention and SDM. The limitation of the study was that the subjects were only categorized into either an expert group or a novice group. The addition of a group of subjects with medium skills would enhance the study. Future research can explore the mechanism by which visual attention training influences SDM by combining event-related potentials (ERPs), eye movement instruments and fMRI.

## Conclusions

Our hypotheses are that female expert basketball players are superior to novice players in MOT ability and SDM and that MOT ability is related to SDM ability. Our results support these hypotheses. (1) The tracking accuracy of expert players was significantly higher than that of novice players, especially when tracking 4–6 targets. As the number of targets increased, accuracy decreased. (2) The accuracy of expert players’ SDM was significantly higher than that of novice players, especially in passing decision-making and shooting decision-making. Expert players exhibited fast and accurate SDM. (3) There was a correlation between MOT ability and SDM ability. The MOT ability of 4–5 targets was positively correlated with passing decision-making, which was statistically significant. The correlation between MOT ability and SDM of expert players was greater and more significant than that of novice players. Having to attend to too many tracking targets (more than 6) interfered with players’ decisions.

## Supporting information

S1 Data (XLSX)

S1 Appendix Decision coding tool table. (DOCX)
