# Supplementary material for: Study on the correlation between basketball players’ multiple-object tracking ability and sports decision-making
Source: PLoS One. 2023 Apr 5;18(4):e0283965. doi: 10.1371/journal.pone.0283965 (PMC10075393; doi:10.1371/journal.pone.0283965)
Supplement: S1 Appendix — (DOCX) [file pone.0283965.s002.docx]

**Decision coding tool table**

| **Decision criteria** | **1 point decision** | **0 point decision** |
| --- | --- | --- |
| Passing | When the pass was passed to the open teammate, the player made a reasonable decision:  (1) A shot is made directly or indirectly, or  (2) Pass to a teammate who is in a better position than the passer. | An unreasonable decision was made when passing the ball:  (1) A pass to a player who is closely guarded, or  (2) There is a defensive player on the passing line, or  (3) The pass was intercepted or blocked, or  (4) No teammates are in a good position on the court, or  (5) The pass is out of bounds. |
| Dribbling | The player makes a reasonable decision while dribbling if it results in:  (1) More room for teammates, or  (2) A chance to score, or  (3) A pick-and-roll for the dribble, or  (4) A drive according to the screen of teammates. | The player made an unreasonable decision while dribbling:  (1) When the defender is in a good defensive position, or  (2) There is no room for dribblers or teammates, or  (3) The dribble goes out of bounds, or  (4) The person who is directly defending is in a good position to defend the dribble, or  (5) No purpose (such as staying in place). |
| Shooting | A shot is a wise decision for a shot player to shoot without being guarded. | The player made an unreasonable decision when shooting:  (1) There are defenders guarding them, or  (2) When one or more defensive players are in a favorable position, or  (3) The ball is stolen while shooting. |

Adapted from: (Romeas, Guldner, & Faubert, 2016); (French & Thomas, 1987).
